# Distribution and colocalization of melatonin 1a-receptor and NADPH-d in the trigeminal system of rat

**DOI:** 10.7717/peerj.6877

**Published:** 2019-05-01

**Authors:** Yifan He, Wenguo Fan, Yue Xu, Yong liang Liu, Hongwen He, Fang Huang

**Affiliations:** 1 Guanghua School of Stomatology, Sun Yat-sen University, Guangzhou, China; 2 Guangdong Provincial Key Laboratory of Stomatology, Sun Yat-Sen University, Guangzhou, China

**Keywords:** NADPH-diaphorase, Mesencephalic trieminal nucleus, Melatonin 1a-receptor, Trigeminal ganglion, Spinal trigeminal nucleus

## Abstract

Melatonin and nitric oxide (NO) are involved in orofacial signal processing in the trigeminal sensory system. The aim of the present study was to examine the distribution of melatonin 1a-receptor (MT1) and its colocalization with nicotinamide adenine dinucleotide phosphate-diaphorase (NADPH-d) in the spinal trigeminal nucleus (STN), the trigeminal ganglion (TG), and the mesencephalic trigeminal nucleus (MTN) in the rat, using histochemistry and immunohistochemistry. Our results show that MT1-positive neurons are widely distributed in the TG and the subnucleus caudalis of the STN. Furthermore, we found that MT1 colocalizes with NADPH-d throughout the TG and MTN, most extensively in the TG. The distribution pattern of MT1 and its colocalization with NADPH-d indicate that melatonin might play an important role in the trigeminal sensory system, which could be responsible for the regulation of NO levels.

## Introduction

Orofacial sensory signals are conducted to the central nervous system by the trigeminal sensory system, which comprises two populations of primary afferent neurons. The majority are located in the trigeminal ganglion (TG), conveying information from mechanoreceptors, thermoreceptors, and nociceptors in the orofacial region. The remainder are situated in the mesencephalic trigeminal nucleus (MTN) in the central nervous system and are involved in proprioception in the masticatory and extrinsic ocular muscles and a subset of periodontal ligament fibers (Stoyanova & Lazarov, 2005; Lazarov, 2002). The second-order neurons are situated in the spinal trigeminal nucleus (STN), which is divided into three subnuclei: in the caudal to rostral direction, caudalis (Vc), interpolaris (Vi), and oralis (Vo) (Sessle, 2000). The Vc is generally considered to play an integral role in the brain stem processing of orofacial nociceptive information and has been regarded as an essential nucleus for orofacial nociception (Sessle, 2011; Chiang et al., 2002). From the STN, the information is conveyed to the higher center.

Melatonin is a functionally versatile neurohormone, produced mainly in the pineal gland in a clear circadian manner, and has a range of activities, including circadian rhythm control and sleep regulation, as well as exerting anti-oxidant and anti-nociceptive effects (Srinivasan et al., 2010; Dehdashtian et al., 2018; Lebda et al., 2018). It is well known that some important effects of melatonin are mediated through activation of specific receptors, which include two membrane-associated receptors (MT1 and MT2) and two other nuclear receptors. Earlier studies found that melatonin receptors are present in various areas of the brain, such as the thalamus, hypothalamus, spinal trigeminal tract, and trigeminal nucleus (Williams, Hannah & Bassett, 1999). Furthermore, Huang et al. (2013) reported that MT1 is expressed in neurons of the TG, MTN, and STN, and its levels are significantly reduced in the Vc during the early stages of orofacial inflammatory pain (Fan et al., 2018).

Nitric oxide (NO) is a neuronal chemical messenger, remarkable for its chemical identity, wide distribution, and broad spectrum of effects (Yeo, 2002). NO is synthesized by nitric oxide synthase (NOS), using L-arginine as a substrate. Nicotinamide adenine dinucleotide phosphate-diaphorase (NADPH-d) is necessary for NOS activity. Thus, NADPH-d histochemistry is a common marker for all three NOS isoforms. Some reports have described NOS expression in the TG and MTN (Stoyanova & Lazarov, 2005; Kolesár et al., 2006). Increasing evidence indicates that NO is implicated in orofacial pain processing (Huang et al., 2013; Fan et al., 2009b).

Melatonin and its receptors are believed to play important roles in nociception and pain modulation (Srinivasan et al., 2010; Fan et al., 2018). Previous studies of body and limb pain showed that melatonin mediates pain via the NO pathway through melatonin receptors (Ulugol et al., 2006; Esposito et al., 2010). The mechanisms by which melatonin influences orofacial pain are poorly understood; melatonin might be involved in the regulation of NO. However, melatonin receptors are incompletely characterized in the trigeminal sensory pathways, and whether melatonin receptors and NOS are coexpressed in these regions remained unknown. Therefore, the aim of the present study was to determine MT1 distribution and colocalization with NOS in the trigeminal system using histochemical and immunohistochemical techniques. Moreover, we attempted to provide a morphological foundation for further research on the role of melatonin and NO in orofacial pain.

## Material and Methods

### Animals and tissue preparation

Five adult female Sprague-Dawley rats (body weight 180–220 g) were used. All of the experimental procedures were approved by the Ethical Review Committee of Guanghua School of Stomatology, Hospital of Stomatology, Institute of Stomatological Research, Sun Yat-Sen University. Animals were deeply anesthetized with 10% chloral hydrate (0.35 ml/100 g body weight, i.p.) and perfused intravascularly with 4% paraformaldehyde in 0.1 M phosphate buffer (pH 7.4). The brainstem and TG were excised, postfixed in the same fixative solution for 6 h, and then stored overnight in 30% sucrose in phosphate buffer at 4 °C until they sank. The brainstem was cut at the level of the Vc (11.30–15.60 mm posterior to bregma) and MTN (9.04–9.76 mm posterior to bregma according to the rat brain atlas). Horizontal sections (25 μm) were cut through ganglia and transverse sections (25 μm) through the brainstem on a cryostat (Leica, Nussloch, Germany). Three series of sections were collected in 0.01 M phosphate-buffered saline (PBS).

### Histological staining methods

The first and second sets of sections for NADPH-d were stained by using 0.1 M Tris buffer (pH 8.0) containing one mM β-NADPH (Sigma-Aldrich, St Louis, MO, USA) and 0.1 mM nitroblue tetrazolium (Sigma-Aldrich, St Louis, MO, USA) with 0.3% Triton X-100. They were kept at 37 °C for 1–2 h. Then the second and the third set were processed for MT1 immunohistochemistry. Following blocking of endogenous peroxidase activity with methanol containing 0.3% hydrogen peroxide (H_2_O_2_) for 20 min, Ultra V Block (LabVision, Fremont, CA, USA) was applied to block nonspecific background staining for 20 min. Sections were then incubated with polyclonal rabbit anti-MT1 serum (1:50; Abbiotec, San Diego, CA, USA) at 4 °C for 18–24 h. For TG and MTN sections, immunofluorescence was used to detect MT1. TG and MTN sections were incubated with TMRITC-conjugated donkey anti-rabbit IgG (1:200; Abcam Corporation, Cambridge, MA, USA) at room temperature for 1 h in the dark. Subsequently, the sections were mounted in weak natural light with glycerol buffer and stored at 4 °C in the dark overnight. STN sections were incubated with biotinylated goat anti-polyvalent (LabVision, Fremont, CA, USA). After incubation in streptavidin peroxidase (LabVision, Fremont, CA, USA), the immunoreactive product was visualized by incubation with diaminobenzidine. Slices were mounted on glass slides, air-dried, gradient-ethanol dehydrated, xylene-cleared (twice, 10 min each), and mounted with neutral gum. For double-labeling of MT1 and NADPH-d, NADPH-d histochemistry was followed by MT1 immunofluorescence/immunohistochemistry staining in the same tissue section. All dilutions and washes between steps were performed using PBS and all steps were carried out at room temperature unless otherwise specified. NADPH-d and MT1 negative control sections were incubated as described but omitting β-NADPH or primary antibodies, resulting in an absence of staining.

### Image analysis

After NADPH-d histochemistry and MT1 immunohistochemistry the sections were examined under a light or fluorescence microscope (Axioskop-40; Carl Zeiss, Hallbergmoos, Germany) equipped with a CCD camera (Carl Zeiss, Hallbergmoos, Germany). Images were processed using Axio Vision 4.5 imaging software (Carl Zeiss, Hallbergmoos, Germany) and Image-Pro Plus software (Media Cybernetics, Silver Spring, MD, USA). Three sections from each series were randomly selected and images were generated using a 200× magnification objective. NADPH-d- and MT1-positive neurons in the TG were subsequently categorized by size as small (<85 μm^2^), medium (>85, <150 μm^2^), and large (>150 μm^2^). The percentage of coexpression was determined from three sections per rat.

## Results

### Expression and colocalization of MT1 and NADPH-d in TG neurons in the rat

**Figure 1:**
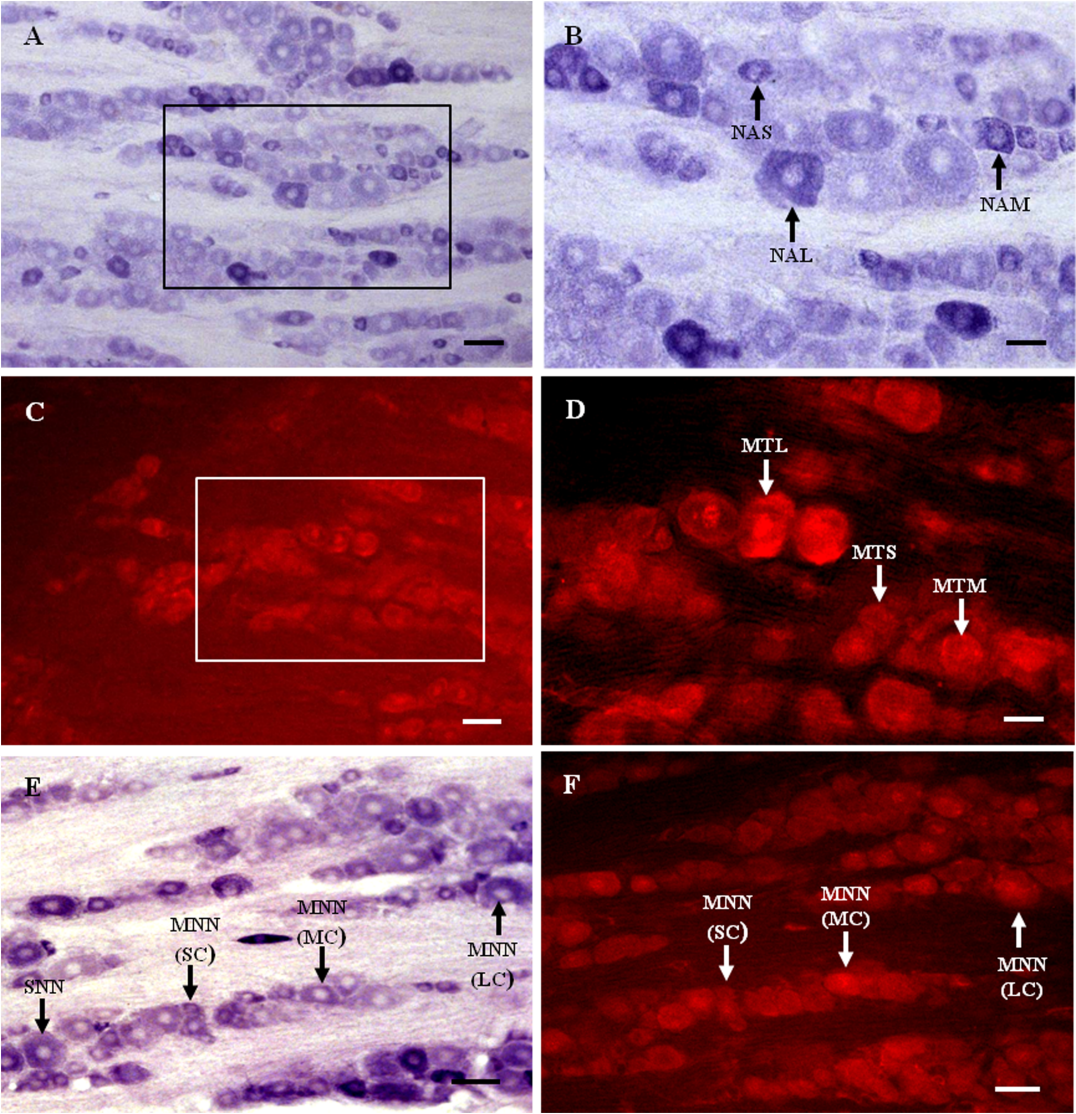
MT1 distribution and colocalization with NADPH-d in TG neurons. (A) and (B) Histochemical staining showing NADPH-positive cells. Neurons of varying sizes (arrows). (C) and (D) Immunofluorescent labeling of MT1-positive neurons. Neurons of varying sizes (arrows). (E) and (F) Coexpression of MT1 and NADPH-d in TG neurons. Double-labeled neurons are distributed widely throughout the TG (arrows). Scale bar: 100 μm (A, C, E, and F); 50 μm (B and D). NAS, small cell of NADPH-positive cell; NAM, medium cell of NADPH-positive cell; NAL, large cell of NADPH-positive cell; MTS, small cell of MT1-positive cell; MTM, medium cell of MT1-positive cell; MTL, large cell of MT1-positive cell; MNN, MT1-positive neurons were double-labeled with NADPH-d; SNN, single NADPH-d-stained cell; SC, small cell; MC, medium cell; LC, large cell.

Nicotinamide adenine dinucleotide phosphate-diaphorase (NADPH-d)-positive neurons were observed in the TG (Figs. 1A and 1B). Neurons of varying sizes (small, 42.4%; medium, 33.5%; large, 23.1%) were mostly round or oval and distributed in clusters throughout the ganglion. There was no prevailing localization of NADPH-d-positive cells in any of the three main parts of the TG (anteromedial, intermediate, posterolateral).

Many MT1-positive neurons were observed in the TG (Figs. 1C and 1D). Neurons of varying sizes (small, 42.9%; medium, 30.8%; large, 26.3%) were mostly round or oval and distributed in clusters throughout the ganglion. There was no prevailing localization of MT1-positive cells in any of the three main parts of the TG (anteromedial, intermediate, posterolateral).

Almost all of the NADPH-d-positive neurons in the TG were double-labeled with MT1 (Figs. 1E and 1F). A few single NADPH-d-stained neurons were diffusely distributed, surrounded by double-labeled neurons. No prevailing localization of double-labeled cells in any of the three main parts of the TG (anteromedial, intermediate, posterolateral) was observed. The proportion of TG neurons coexpressing MT1 and NADPH-d was 84.7% (1554/1835).

### Expression and colocalization of MT1 and NADPH-d in MTN neurons in the rat

**Figure 2:**
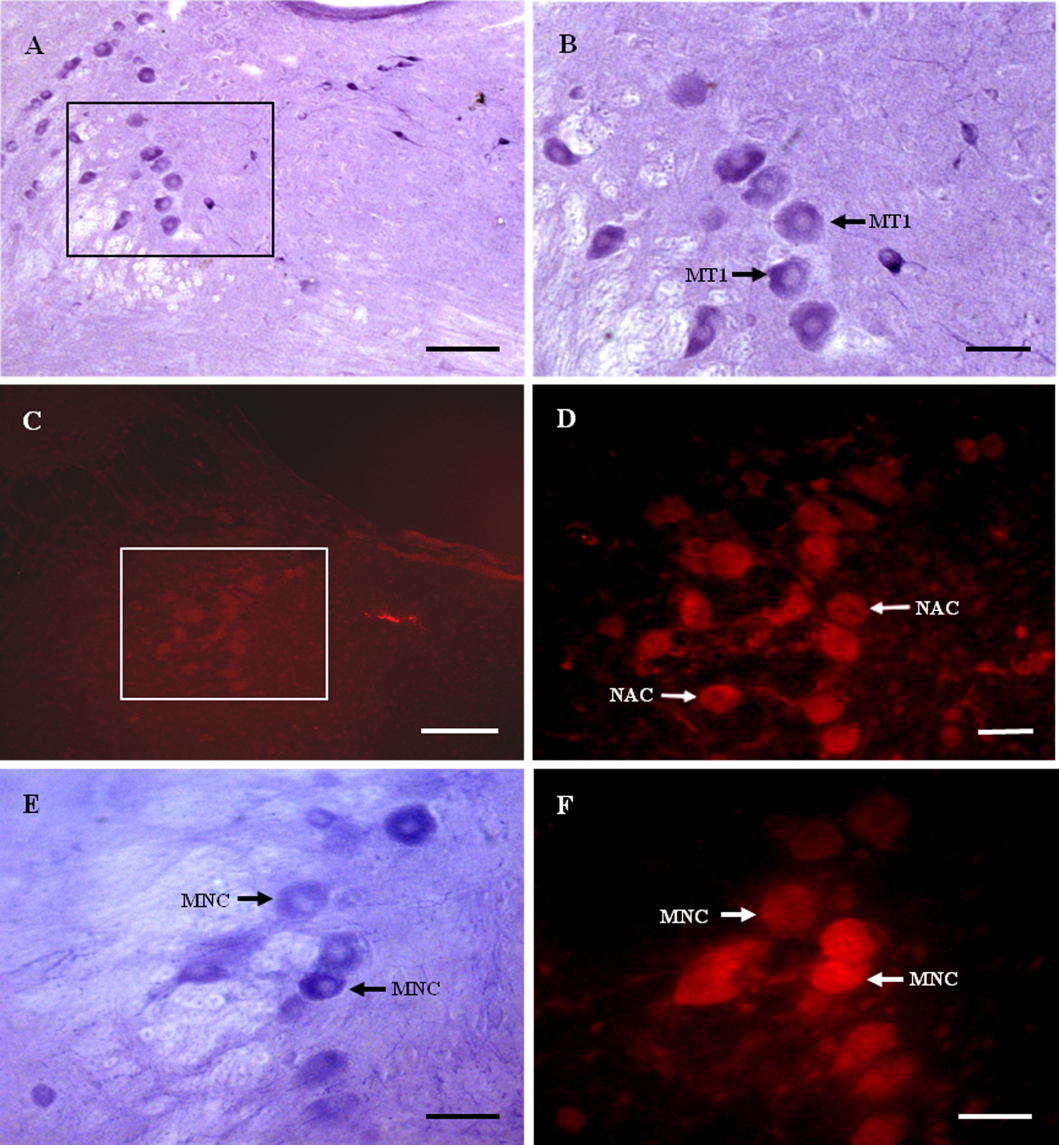
MT1 distribution and colocalization with NADPH-d in MTN neurons. (A) and (B) Histochemical staining showing NADPH-positive cells (arrows). (C) and (D) Immunofluorescent labeling of MT1-positive neurons (arrows). (E) and (F) Coexpression of MT1 and NADPH-d in MTN neurons. Most NADPH-d-positive neurons in the MTN coexpressed MT1 (arrows). Scale bar: 100 μm (B, D, E, and F); 50 μm (A and C). MT1, MT1-positive cell; NAC, NADPH-positive cell; MNC, MT1-positive neurons were double-labeled with NADPH-d.

Nicotinamide adenine dinucleotide phosphate-diaphorase-positive neurons were observed in the MTN (Figs. 2A and 2B). MT1-immunoreactive neurons were observed in the MTN (Figs. 2C and 2D). The majority of MT1 expressing cells were large and most of them were spherical or oval. Some MT1-positive fibers were observed between the neurons.

**Figure 3:**
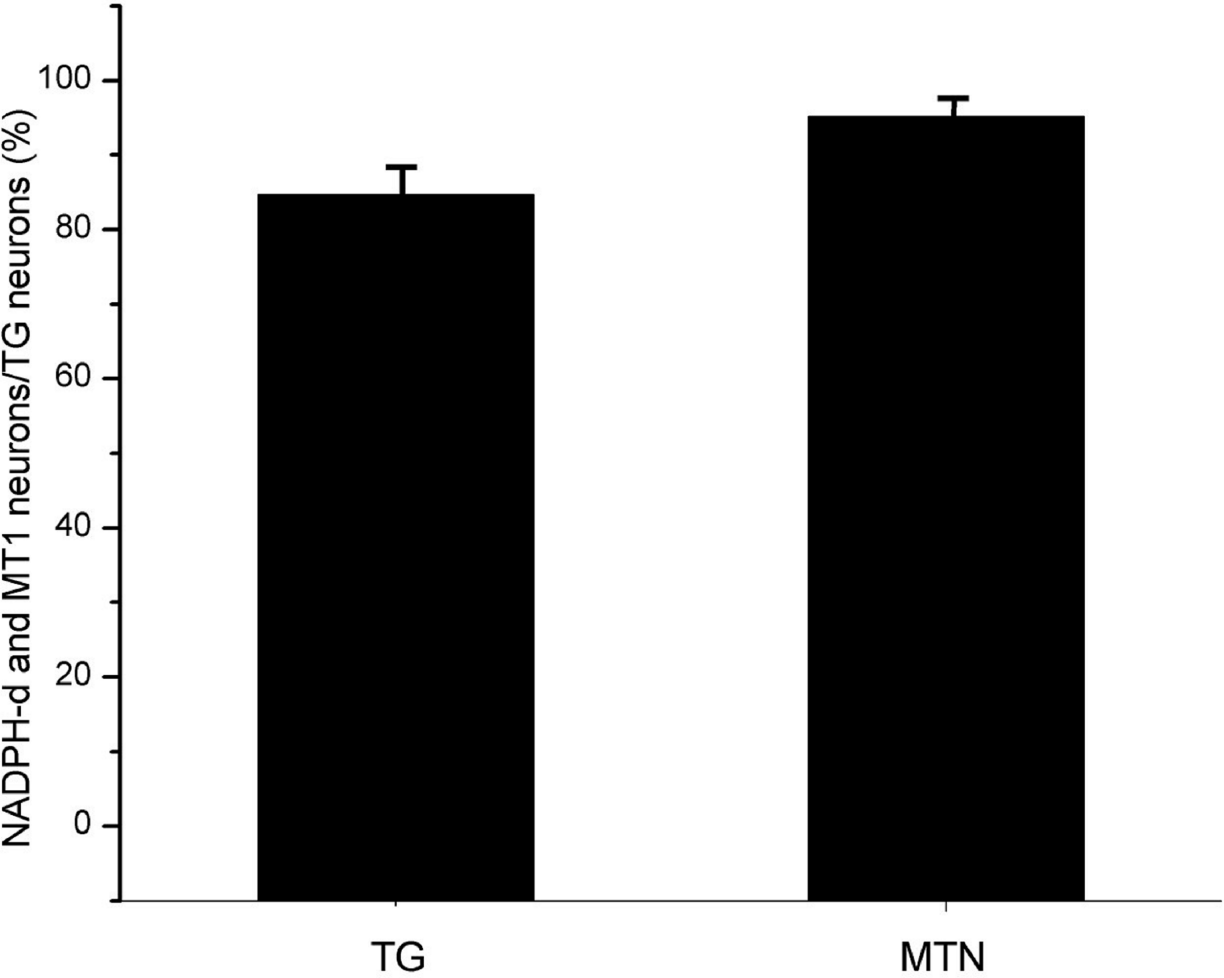
The proportion of coexisting neurons MT1 and NADPH-d in TG and MTN. TG, The proportion of TG neurons coexpressing MT1 and NADPH-d; MTN, The proportion of MTN neurons coexpressing MT1 and NADPH-d.

Most NADPH-d-positive neurons in the MTN coexpressed MT1 (Figs. 2E and 2F). There were considerably fewer NADPH-d-positive neurons in the MTN than in the TG, and 95.1% (116/122) were double-stained for NADPH-d and MT1 (Fig. 3).

### Expression and colocalization of MT1 and NADPH-d in STN neurons in the rat

**Figure 4:**
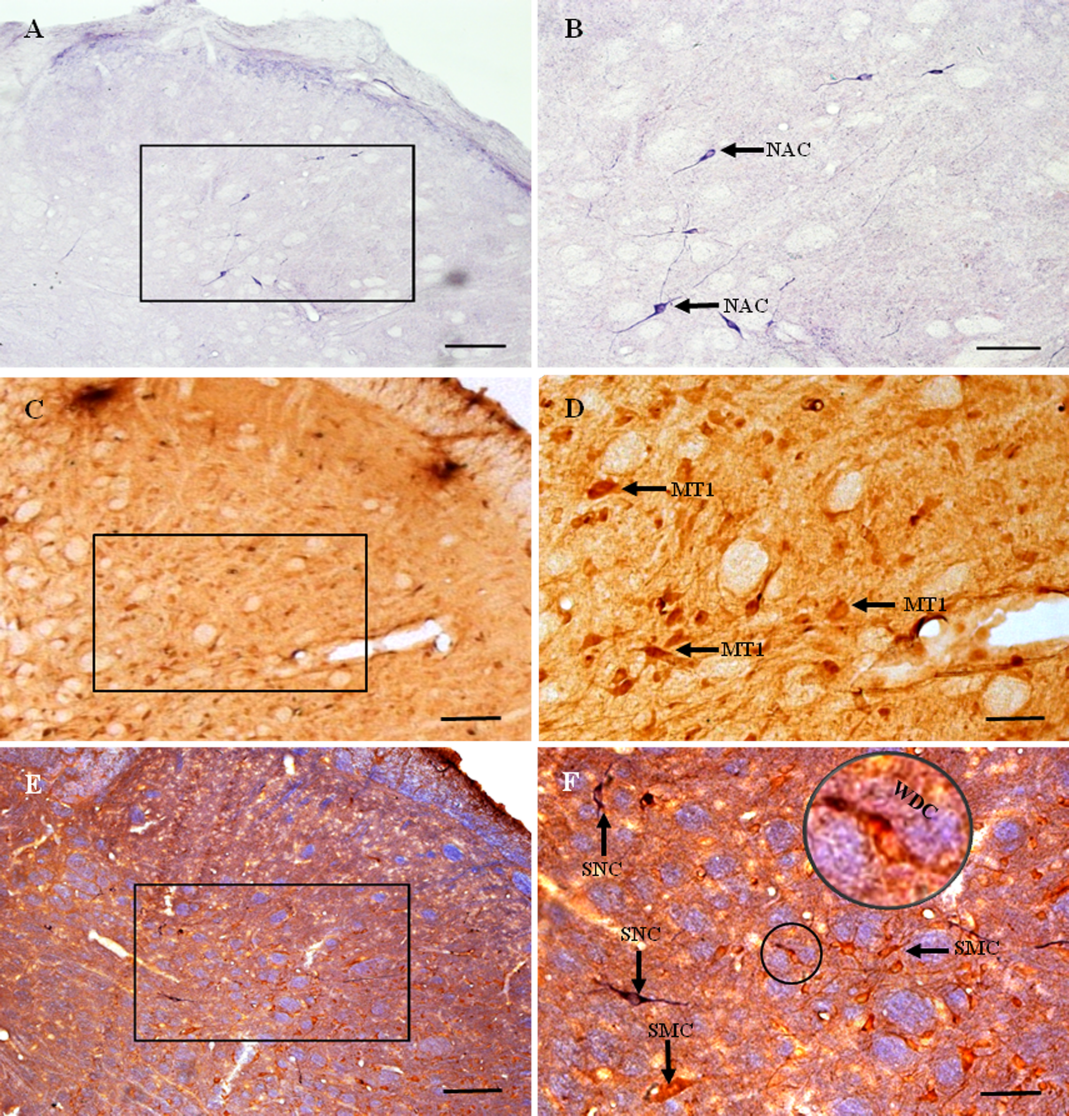
MT1 distribution and colocalization with NADPH-d in STN neurons. (A) and (B) Histochemical staining showing NADPH-d-positive cells (arrows). (C) and (D) Immunohistochemical staining showing MT1-positive neurons (arrows). (E) and (F) Coexpression of MT1 and NADPH-d in Vc neurons. Most MT1-expressing neurons in the Vc were not coexpressed NADPH-d, a few of ones were weakly double-stained cells for MT1 and NADPH-d. Scale bar: 100 μm (A, C, and E); 50 μm (B, D, and F). MT1, MT1-positive cell; NAC, NADPH-d-positive cell; WDC, weakly double-stained cell for MT1 and NADPH-d; SNC, single NADPH-d-stained cell; SMC, single MT1-positive cell.

Nicotinamide adenine dinucleotide phosphate-diaphorase-positive cells and fibers were distributed throughout the STN (Figs. 4A and 4B). Most were elongated or bipolar. NADPH-d-positive cells and fibers were visible in laminae I–IV in the STN.

MT1-positive neurons were distributed extensively throughout the STN (Figs. 4C and 4D). Most were polygonal cells, with a few spindle-shaped cells localized sparsely among them. MT1-stained neurons appeared brown and staining was particularly intense in the perinuclear region. In deeper layers, staining around the nucleus was more prominent. Most MT1 expressing neurons lay in laminae II–IV. MT1 staining was more intense in the Vc than in the Vi and Vo.

There was no clearly double-labeled neuron for MT1 and NADPH-d in the STN, and only a few neurons were weakly double-stained (Figs. 4E and 4F). There were many more MT1-positive than NADPH-d-positive neurons, mostly in laminae II–IV.

## Discussion

### Expression of MT1 in the trigeminal sensory system

Melatonin receptors have been studied for several decades. Studies using autoradiography (Williams, Hannah & Bassett, 1999; Thomas et al., 2002), in situ hybridization (Thomas et al., 2002), and the reverse transcription polymerase chain reaction (Uz et al., 2005; Zahn et al., 2003) have shown that melatonin receptors are present in various areas of the brain and spinal cord, including the thalamus, hypothalamus, spinal trigeminal tract, and trigeminal nucleus. MT1 belongs to a subfamily of G protein-coupled receptors. Zahn et al. (2003) and Thomas et al. (2002) reported that MT1 is the major receptor in the central nervous system.

However, the tissue and cellular distribution of MT1 in the trigeminal sensory system is unclear. The present study demonstrates that MT1 is expressed widely in the TG, Vc, and MTN. A previous study from our research group revealed a significant upregulation of NADPH-d in the TG and downregulation of MT1 in the Vc during the early stages of orofacial inflammatory pain (Huang et al., 2013). Also, Wang et al. (2012) reported that inflammation of the temporomandibular joint causes a downregulation of MT1 expression in the ipsilateral Vc. These results suggest that MT1 plays a role in orofacial pain modulation. In the spinal somatosensory system, melatonin has been shown to exert strong antinociceptive effects (Wilhelmsen et al., 2011; Ambriz-Tututi et al., 2009; Huang et al., 2018). Melatonin was found to interfere with the N-methyl-D-aspartic acid (NMDA)-mediated glutamatergic component of pain transmission in the spinal cord, known as the “wind-up” effect, by acting on melatonin receptors (Laurido et al., 2002; Mondaca et al., 2004; Wen et al., 2016). However, most reports implicated that MT2 instead of MT1 is involved in the mediation of antinociceptive actions of melatonin (Ambriz-Tututi & Granados-Soto, 2007; Arreola-Espino et al., 2007), because its analgesic effect was blocked by selective MT2 antagonists. However, a lack of selective MT1 antagonists makes it difficult to rule out a role of MT1 (Comai & Gobbi, 2014). It is known that MT1 and MT2 have either opposing or complementary functions (Mor et al., 2010). Therefore, the possibility that both MT1 and MT2 take part in spinal somatosensory modulation cannot be dismissed.

The Vc has many anatomical and functional similarities with the spinal dorsal horn; it has a laminated structure and contains several morphologically distinct neuronal types that are generally comparable with those described in the spinal dorsal horn (Sessle, 2000). The TG innervates mainly nociceptors, mechanoreceptors, and thermoreceptors in the face, oral cavity, and nasal cavity (Davies, 1988). We found that MT1 immunoreactivity was most extensive in the Vc and TG, which are involved in orofacial pain processes. We therefore speculate that MT1 plays an important role in orofacial pain processing. Further research is required to identify the precise mode of action of MT1 in pain regulation.

### Expression of NO/NOS in the trigeminal sensory system

To date, three distinct isoforms of NOS have been identified, namely, constitutive neuronal NOS (nNOS), endothelial NOS (eNOS), and inducible NOS (iNOS) (Nathan & Xie, 1994). In the present study, we described the distribution of NADPH-d without determining which isoforms of NOS were involved. Our results agree with those from other studies (Kolesár et al., 2006; Fan et al., 2008, 2009a). It should also be noted that most of the Vc neurons involved in orofacial nociception do not synthesize NO under normal conditions, but are modulated by NO, which diffuses from adjacent NOS-positive neurons (Yeo, 2002). Accumulating evidence implicates that NO is involved in spinal pain processing (Cury et al., 2011; Callsen-Cencic et al., 1999). Furthermore, NO is a significant neuromodulator in orofacial pain. Our previous study showed that chronic pulp inflammation markedly increased NADPH-d activity in the TG and the numbers of NADPH-d-positive neurons in the Vc (Fan et al., 2009a). This evidence indicates that NO may have a pronociceptive effect.

Other studies support a pronociceptive effect of NO in orofacial pain processing. Sugiyama et al. (2013) reported NO metabolites and nNOS-immunoreactive neurons increase in the TG in an ectopic orofacial pain model induced by inferior alveolar nerve transection, and nNOS inhibitors alleviate mechanical allodynia in this model. Borsani et al. (2010) showed that a significant increase in nNOS levels was found in the TG in an experimental murine model of inflammatory orofacial pain. Purinergic receptor antagonists can modulate the NO system and influence orofacial pain transmission. However, Rodella et al. (2000) reported that there are fewer NADPH-d-positive neurons in the TG in trigeminal hyperalgesia induced by diabetes. Several studies also agree that NO is essential in orofacial pain processing (Almeida & Duarte, 2008; Fávaro-Moreira et al., 2009; Schütz, Andersen & Tufik, 2004), but its exact role and mechanism remain to be elucidated.

### Colocalization of MT1 and NADPH-d in the trigeminal sensory system

Primary sensory fibers innervating the orofacial region are derived from neurons of the TG and MTN (Lazarov, 2000). The TG innervates mainly mechanoreceptors, thermoreceptors, and nociceptors in the orofacial region (Davies, 1988; Huang et al., 2018), while the MTN mostly innervates proprioceptors in the orofacial region (Walberg, 1984; Byers et al., 1986). Our results demonstrate colocalization of NADPH-d and MT1 in the TG and MTN, with a ratio of 84.7% and 95.1%, respectively. This suggests that NO and melatonin might interact in the trigeminal primary sensory system.

We found few neurons coexpressing MT1 and NADPH-d in the Vc; colocalization of NADPH-d and MT1 was more prominent in the TG and MTN. This suggests that MT1 and NADPH-d might interact in the trigeminal sensory system, particularly in the periphery. Interaction of MT1 and NADPH-d in the STN cannot be excluded by their low rate of coexpression, however, because most of the Vc neurons involved in orofacial nociception are modulated by NO diffused from adjacent NOS-positive neurons (instead of NO synthesized by themselves) (Yeo, 2002). To date, there have been no reports on the colocalization of MT1 and NADPH-d in the trigeminal sensory system. In the somatosensory system, inflammatory pain experiments have shown that melatonin is involved in pain modulation by modulating NO levels (Schütz, Andersen & Tufik, 2004; Mantovani et al., 2006). Hernández-Pacheco et al. (2008) demonstrated melatonin is involved in spinal pain modulation in the formalin test, through the NO–cyclic GMP–protein kinase G–K^+^ channel pathway. As mentioned above, melatonin exerts its effect on spinal cord nociceptive transmission through the NMDA receptor-dependent pathway (Laurido et al., 2002). An NMDA receptor-dependent NO generating pathway has also been implicated in the spinal transmission of nociceptive information (Stanfa, Misra & Dickenson, 1996). Furthermore, intrathecal administration of NMDA has a bidirectional effect on the C-fiber-evoked activity of Vc neurons (Luccarini, Sessle & Woda, 2001). Together, the data suggest that NMDA receptors might be involved in the interaction between melatonin and NO.

Few studies have examined the relationship between melatonin and NO in the orofacial sensory system, although some indirect evidence suggests that there is an interaction between them. NO, a potential source of free radicals, may play an important role in inflammatory and neuropathic pain (Kim et al., 2010; Tang et al., 2009). Melatonin inhibits the activity of NOS and has NO and peroxynitrite scavenging activity (Aydogan, Yerer & Goktas, 2006; 54). Chang et al. (2000) found that melatonin markedly inhibits NADPH-d and nNOS expression in the hypoglossal nucleus following hypoglossal nerve transection. Sarti et al. (2013) reported that at a concentration of ~10^−9^ M, melatonin transiently increases synthesis of the nNOS protein and NO oxidation products in cultured HaCaT cells; these results are similar to those of Arese et al. (2012). Furthermore, trigeminal nerve fiber activation is considered to be a key factor in the pathogenesis of migraine. Melatonin might exert its effect on the treatment of migraine by modulation of NO levels (Peres, 2005; Eli & Fasciano, 2006) and NO/NOS may be involved in the circadian rhythm (Amir, Robinson & Edelstein, 1995; Kunieda et al., 2008). Melatonin, a hormone regulating the circadian rhythm, exerts its phase shifting effect by activation of NO synthesis (Starkey, 1996). It is likely that melatonin interacts with NOS, because NO is a short-lived free radical. MT2 has been reported to mediate phase shift effects of melatonin (Dubocovich & Markowska, 2005), but current knowledge does not allow us to rule out a possible role of MT1 receptors in the phase shift of SCN neuronal activity due to melatonin (Comai & Gobbi, 2014). The specific melatonin receptor that is involved in these effects has not yet been identified and there is insufficient evidence on the role of MT1 and NOS. In the present study, the colocalization and special distribution pattern of NADPH-d and MT1 in the trigeminal sensory system imply that melatonin might contribute to the orofacial sensory processing by interacting with NOS through MT1. Much is known about melatonin and NOS/NO signaling pathways, but how melatonin acts on NOS/NO in orofacial sensory processing remains to be explored.

In brief, MT1 is widely expressed in the trigeminal sensory system, particularly in the TG and Vc. Furthermore, colocalization with NADPH-d was observed, most extensively in the TG and MTN, suggesting that an important interaction exists between melatonin and NO in these nuclei. Although the physiological roles of melatonin and NO in the nervous system are not yet fully elucidated, our results provide morphological evidence for the involvement of melatonin in orofacial sensory modulation by the regulation of NO levels.

## Supplemental Information

10.7717/peerj.6877/supp-1 Supplemental Information 1 Raw data.

## Additional Information and Declarations

### Data Availability

The following information was supplied regarding data availability: The raw data is available in a Supplementary File.
